# Prospects and Opportunities Soon to Be Encountered

**Published:** 1919-09-06

**Authors:** 


### MEDICAL RESEARCH AND THE STUDENT.

### Prospects and Opportunities Soon to be Encountered.

Since the cessation of hostilities there have been few more talked of subjects than medical research, particularly that with direct bearing upon matters of public health and the possible activities of the new Ministry of Health. In a brief space, even were it necessary, it is impossible to outline the numerous organised investigations which have been conducted throughout the war period. In this Journal we have repeatedly given summaries of their work as, and before, the results were officially published and for their details we would refer the reader to our recent issues. At the moment it is our object to point out some of the fundamental principles on which this type of work must be carried on, and to suggest to those in the early stages of, or now entering, the medical profession a few of the preliminary steps to success in research if their natural bent be in that direction.

Scientific medicine can now boast of so many subdivisions, each sufficiently developed to tend to claim the undivided attention of the individual, that it has already become obvious the only truly efficient way in which progress can be made is through the agency of organised teamwork on the part of a set of thoroughly competent authorities, working sympathetically with each other and with the freest facilities for intercommunication. Under each of these authorities must act a series of assistants to collect, correlate, and record the results of work carried out at their senior's suggestion, and also to develop the minor sub-sections on their own account. In any instance, there are mountains of detail, small technical and laboratory difficulties, administrative requirements of clinics, etc., to be dealt with before the end-product, the result of medical research as a series of determined facts, can be produced by the team. In other words, there is required what practically amounts to a research "service," with proper ranks, assigned duties, and senior controlling officers. Already we have seen the early possibilities under direct Army control, and the example of the excellent work of the Medicine Research Committee. The suggested developments in our greater hospitals show abundant signs of future success, and it now remains for the Ministry to foster this spirit of organised effort to the utmost.

In this country at the moment we have before us the problem of imposing the revolution, which general adoption of this principle must involve, upon a host of time-honoured professional and institutional customs. Also there is the question of ways and means, both for the individual and the organisation.

In the first place, we believe that it is extremely difficult, if not impossible, if true maximum efficiency is to be attained, for the medical men concerned in such work to divide their attention between it and the monetary side of private specialist practice. As position, personal reputation, and experience become greater, the essential causes of this objection tend to disappear, but even then it is certain that the investigational work of a man, however eminent, must tend to decline in quality when hampered by divergent interests. Much more strongly does this occur as we descend in our "service" scale until it becomes obvious that, as our guiding authorities urge and develop research schemes, they must take the greatest care that there are funds forthcoming, sufficient not only for the equipments and institutions concerned, but adequate living remuneration for the number of skilled men required to carry out the work. The point we would urge is that, quite apart from consideration of fairness or otherwise to the individual scientist or doctor and the just reward for his labours, there is only one way in which the best investigations can be organised and that is by granting them the undivided attention of their instigators. This can never occur generally throughout the country unless full and sufficient endowment of these schemes be granted.

The signs of the times suggest that medical and institutional authorities are not only realising, but will act upon this principle in future. In that case we may expect to see a number of our promising younger medical men, many of whom, though having every instinct of the research student, have in the past been forced by circumstances and lack of reasonable opportunity into the most routine forms of general practice, entering instead upon investigational work. At all times there are, and have been, a high percentage of those "walking" our hospitals who are strongly attracted by the fringes of medical research, upon which, even during their novice days, they have opportunity to touch, and it has been more than disappointing to see the number of promising men who annually forsake this early ideal. We need research; to cultivate it, we need also to cultivate the spirit in the younger generation, and it is one of the most hopeful of post-war indications that our medical schools are realising their duty in this direction. True, it may be some time before the effect is definite and readily observable, but we hear of many developments and innovations for the new session about to open, and hospital staff committees apparently intend to pursue the policy of linking and teamwork, not only in early teaching, but in post-graduate measures as well. In a few months the actual working of these plans will give yet more indication for the future, but even at the moment it is probable that a man who has, realising its limitations in a financial sense, the natural taste and desire for the career of a true investigating scientist, will find that the future presents no better path, nor one likely to be indirectly of more value to his fellow-countrymen, than that of the medical profession.

